# Type A insulin resistance syndrome misdiagnosed as polycystic ovary syndrome: a case report

**DOI:** 10.1186/s13256-019-2304-4

**Published:** 2019-11-27

**Authors:** Lu Lin, Cunren Chen, Tuanyu Fang, Daoxiong Chen, Kaining Chen, Huibiao Quan

**Affiliations:** Department of Endocrinology, Hainan General Hospital, No. 19, Xiuhua Road, Xiuying District, Haikou City, Hainan Province China

**Keywords:** Type A insulin resistance syndrome, Insulin receptor gene mutations, Hyperinsulinemia, Polycystic ovary syndrome

## Abstract

**Background:**

Type A insulin resistance syndrome, one type of the hereditary insulin resistance syndromes, is a rare disorder. Patients with type A insulin resistance syndrome are nonobese and demonstrate severe hyperinsulinemia, hyperandrogenism, and acanthosis nigricans. The clinical features are more severe in affected females than in males, and they mostly become apparent at the age of puberty. In many cases, when severe insulin resistance is covered up by other signs or symptoms of type A insulin resistance syndrome, patients are often easily misdiagnosed with other diseases, such as polycystic ovary syndrome.

**Case presentation:**

Our patient was a 27-year-old Han Chinese woman who sought treatment because of a menstrual disorder and hirsutism. Tests showed that her levels of insulin and testosterone were elevated, and gynecological color Doppler ultrasound suggested multiple cystic changes in the bilateral ovaries. After a diagnosis of polycystic ovary syndrome was made, pulsatile gonadotropin-releasing hormone therapy and metformin were administered, but the patient’s symptoms did not improve in 1 year of follow-up. Considering that the previous diagnosis might have been incorrect, venous blood samples were collected from the patient and her relatives for genetic analysis. Subsequently, using Illumina sequencing, it was found that the proband, her father, and two brothers all had the c.3601C>T heterozygous missense mutation in exon 20 of the insulin receptor gene. The diagnosis was corrected to type A insulin resistance syndrome, and the patient’s treatment was modified.

**Conclusion:**

We report a case of a young woman with type A insulin resistance syndrome that was misdiagnosed as polycystic ovary syndrome. We discuss the causes, clinical features, diagnosis, and treatment of type A insulin resistance syndrome to improve the recognition of the disease and reduce its misdiagnosis. Female patients with high androgen levels and severe hyperinsulinemia should be considered for the possibility of hereditary insulin resistance syndromes (such as type A insulin resistance syndrome). Gene sequencing helps in making an early diagnosis and developing a targeted treatment strategy.

## Background

Type A insulin resistance syndrome (TAIRS) is rare in the clinic, with an incidence of approximately 1 in 100,000 [1]. The clinical manifestations of the disease are complex, and clear diagnosis is dependent on gene sequencing. Clinicians generally lack an understanding of the disease, which results in many patients being misdiagnosed and incorrectly treated. This paper reports a case of a patient with TAIRS who was misdiagnosed with polycystic ovary syndrome (PCOS). Gene sequencing was performed to screen for mutations in the proband and her first-degree relatives. The relevant literature is reviewed and summarized.

## Case presentation

The patient, a 27-year-old Han Chinese woman, was admitted to our department on July 13, 2017, due to a menstrual disorder of 4 years’ duration. She had experienced a reduction in menstrual frequency 4 years prior, without any obvious cause, with the longest period cycle being up to 4 months and with a decreased amount of menstruation. At the same time, black, coarse hair appeared in her mandible, medioventral line, and areola, and her pubic hair became thicker, accompanied by breast shrinkage. The patient also easily became hungry. No hoarse voice or facial acne appeared. The patient was treated with progesterone at a local hospital, and withdrawal bleeding happened after the treatment. The symptoms recurred after withdrawal of the medicine. No special past history was reported. The patient had menarche at the age of 15, with a period cycle of 28–120 days and a menstrual period duration of 4–5 days. Her most recent menstruation was on June 1, 2017 (after the administration of progesterone). The patient denied a family history of similar diseases. An outpatient test revealed that the patient’s testosterone level was 4.07 nmol/L (reference range, 0.43–2.06 nmol/L). Gynecological color Doppler ultrasound showed bilateral ovaries approximately 44 mm × 23 mm in size on the left side and approximately 50 mm × 26 mm on the right side and more than 10 small follicle echoes inside both ovaries. The diagnosis suggested multiple cystic changes in the bilateral ovaries. Adrenal computed tomography showed that the medial limb of the left adrenal gland was enlarged, with a high possibility of proliferation. The patient’s admission physical examination showed that her body temperature was 36.8 °C, pulse 100 beats/minute, respiration 20 breaths/minute, and blood pressure 119/79 mmHg. The patient’s body mass index (BMI) was 16.89 kg/m^2^. She had a normal appearance, with no facial acne, full moon face, or buffalo hump and no signs of acanthosis nigricans. Heart, lung, and abdominal examinations showed no abnormalities. Black, coarse hair was observed on the patient’s upper lip, lower jaw, chest, bilateral areolas, and medioventral line of the lower abdomen. The bilateral breasts were symmetrical, and the nipples were slightly invaginated. The pubic hair was thick, and the clitoris was hypertrophied. The Tanner stage was B3P5. The laboratory test results for six gonadal hormones were as follows: luteinizing hormone (LH), 4.96 IU/L (reference range, 1.8–11.78 IU/L); follicle-stimulating hormone (FSH), 2.04 IU/L (3.03–8.08 IU/L); estradiol, 129.0 pmol/L (77.1–921.2 pmol/L); progesterone, 1.03 nmol/L (< 0.318–0.954 nmol/L); prolactin, 494.0 mIU/L (108.8–557.1 mIU/L); and testosterone, 3.40 nmol/L. The patient’s cortisol level was normal, and the secretion rhythm was present and could be inhibited by an overnight 1-mg dexamethasone suppression test. The oral glucose tolerance test (OGTT) result reached the diagnostic criteria for diabetes, and the levels of synchronized insulin were significantly elevated (0 minutes, 701.70 pmol/L; 120 minutes, > 6945 pmol/L). The patient’s dehydroepiandrosterone concentration was 7.44 ng/ml (0.80–10.50 ng/ml); dehydroepiandrosterone sulfate was 27.20 nmol/L (18.00–144.00 nmol/L); 17-hydroxyprogesterone (17-OHP) was 1.44 ng/ml (0.05–1.02 ng/ml); and human anti-Müllerian hormone was 16.40 ng/ml (2.80–6.30 ng/ml). Karyotype analysis showed a karyotype of 46,XX. The patient’s liver and kidney function, blood lipids, and thyroid function were normal. A recheck of the gynecological color Doppler ultrasound data showed a polycystic state of both ovaries and small cysts in the cervix. No abnormality was revealed by urinary system and abdominal color ultrasound. The patient was diagnosed with PCOS with type 2 diabetes. Considering that she was a nonobese patient with PCOS and had an LH/FSH ratio of 2.43 with an abnormal LH pulse frequency, pulsatile gonadotropin-releasing hormone was administered via a pump, which can continuously infuse gonadorelin subcutaneously to restore hypothalamic-pituitary-gonadal axis function and promote menstrual cycle recovery with ovulation. At the same time, metformin was administered to improve insulin sensitivity. After 1 year of follow-up at monthly intervals, the testosterone level of the patient remained above normal and fluctuated between 2.37 and 2.63 nmol/L. Her menstrual disorder, hirsutism, and other symptoms did not improve. Reexamination on July 19, 2018, showed testosterone of 2.74 nmol/L and fasting insulin of 464.50 pmol/L. We considered that the previous diagnosis might have been incorrect, and the patient was hospitalized again on July 31, 2018. A supplementary medical history was collected and showed that the patient’s parents were not close relatives and that the father had type 2 diabetes and was treated with metformin. The mother and three brothers denied a history of diabetes. OGTT was performed on the patient and her mother, and a steamed bread meal test was performed on the father; the results are listed in Table 1. A human insulin receptor (INSR) antibody was purchased from Wuhan Huamei Bioengineering Co., Ltd. in Hubei Province, and an enzyme-linked immunosorbent assay (using a KHB ST-360 microplate reader) showed negative results for serum INSR antibodies.

**Table 1:** Clinical data of a patient with type A insulin resistance syndrome and her parents

Subject	Age (years)	OGTT										Reference range
		FPG (mmol/L)					INS (pmol/L)					
		0 minutes	30 minutes	60 minutes	120 minutes	180 minutes	0 minutes	30 minutes	60 minutes	120 minutes	180 minutes	
Patient	28	4.20	14.40	18.40	14.00	10.50	508.40	5636.00	> 6945	> 6945	> 6945	FPG: 3.9–6.1
Father	64	7.30	13.50	18.50	17.30	12.20	264.40	547.50	991.00	1056.00	908.80	Fasting INS: 17.8–173
Mother	56	4.90	8.90	8.70	7.20	5.20	70.96	736.40	977.90	1006.00	293.30	

*FPG* fasting plasma glucose, *INS* fasting serum insulin

**Fig. 1:**
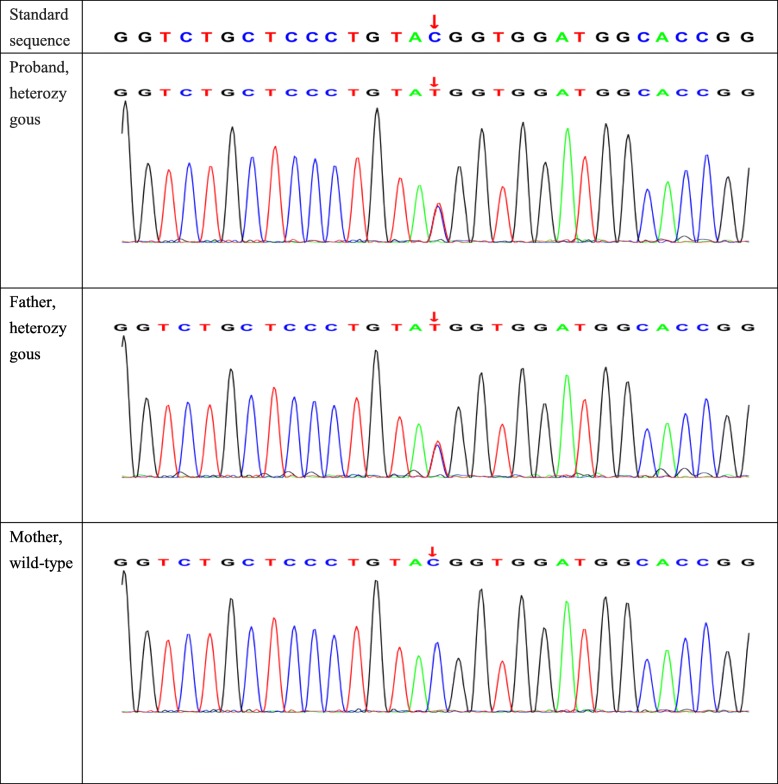
Sequencing results of the *INSR* gene in the proband and her parents

**Fig. 2:**
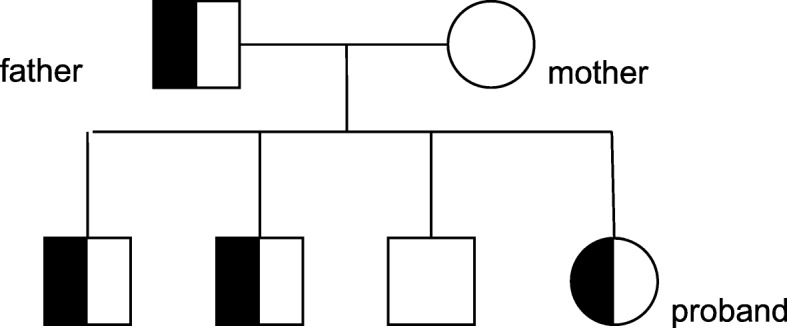
Pedigree of the type A insulin resistance syndrome family

After obtaining consent of the patient and her family and a signed informed consent form, 5 ml of peripheral venous blood was collected from the patient and her parents, and genomic DNA was extracted using an ultrasound method. The xGen Exome Research Panel version 1.0 (Integrated DNA Technologies, Coralville, IA, USA) was employed for whole-genome exon capture, and the obtained DNA fragments were then subjected to high-throughput sequencing using the NovaSeq 6000 system (Illumina, San Diego, CA, USA). The raw image files were processed using bcl2fastq (Illumina) to generate raw sequencing data. Low-quality reads, with a quality score less than 20, were filtered out. The resulting sequences were aligned with the human genome reference sequence (hg19), provided by the National Center for Biotechnology Information, using the Burrows-Wheeler Aligner. Single-nucleotide polymorphisms, insertions, and deletions were analyzed in the sequences using SAMtools and Pindel. The data interpretation rules referred to the classification criteria and guidelines for genetic variations of the American College of Medical Genetics and Genomics. The results of the genetic testing showed that the patient and her father had a heterozygous missense mutation, c.3601C>T, p.Arg1201Trp, in the *INSR* gene (NM_000208) (Fig. 1). The gene sequence of the mother was normal at the same locus. Further sequencing of the above locus in the patient’s three brothers revealed that two of them also had the mutation at the same site; however, clinical data of the three brothers have not been obtained yet. The family pedigree is shown in Fig. 2.

Comprehensive evaluation of the patient’s clinical manifestations, laboratory test results, and gene sequencing led to a clear diagnosis of TAIRS. The treatment regimen was changed to pioglitazone (30 mg once daily) and spironolactone (20 mg twice daily). After 2 months, the patient’s blood potassium level was 4.57 mmol/L, testosterone was 2.91 nmol/L, 0-minute insulin was 299.40 pmol/L, and 120-minute insulin was > 6945 pmol/L. The patient still had no menstruation and no improvement of her hirsutism symptoms. The dosage of spironolactone was adjusted to 40 mg twice daily. On March 20, 2019, the patient’s blood potassium was 4.65 mmol/L, testosterone was 3.08 nmol/L, and fasting insulin was 411.00 pmol/L. The treatment regimen was modified to metformin (0.5 g twice daily), pioglitazone (30 mg once daily), and flutamide tablet (0.0625 g once daily).

## Discussion and conclusion

Insulin resistance (IR) is clinically defined as a decrease in the sensitivity of the body to exogenous or endogenous insulin and a glucose uptake and use disorder of the target organs of insulin [2]. IR is usually caused by a combination of genetic predisposition and obesity, whereas extremely severe IR can also be observed in very few nonobese individuals. The latter usually carry identifiable single-gene defects, and a definitive diagnosis can provide these patients with individualized treatment options and further genetic counseling.

Genetic mutations can cause impairments in insulin signaling pathways, leading to severe IR, the most important component of which is INSR. INSR is a covalent dimer composed of two α-subunits and two β-subunits, linked by disulfide bonds [3]. The human *INSR* gene is located on autosome 19p13.2–13.3 and has a total length of more than 170 kb, including 22 exons and 21 introns. Exons 1–11 encode the α-subunit, and exons 12–22 encode the β-subunit [4]. Currently, more than 150 *INSR* gene mutations have been identified that can cause hereditary IR syndrome. Mutation types include missense mutations, nonsense mutations, deletions, insertions, and complex rearrangements. The effects of *INSR* gene mutations on the receptor function are divided into the following five categories [5]: (1) reduction in INSR biosynthesis, (2) impaired transportation of INSR to the cell surface, (3) decreased affinity of insulin binding, (4) inhibition of tyrosine kinase activity, and (5) accelerated degradation of INSR. Gene mutations within the β-subunit, some of which are also detected in patients with type 2 diabetes, usually cause a loss or attenuation of tyrosine kinase activity [5, 6].

There are three main types of hereditary IR syndromes, namely Donohue syndrome, Rabson-Mendenhall syndrome, and TAIRS. These three syndromes are considered different manifestations of the same continuous disease spectrum. Donohue syndrome is the most severe, with affected children usually dying before the age of 2 years. TAIRS is at the least severe end of the disease spectrum and is usually not life-threatening. Patients with TAIRS are nonobese and demonstrate severe hyperinsulinemia, hyperandrogenism, and acanthosis nigricans [7]. The incidence of TAIRS is approximately 1 in 100,000. Because female patients demonstrate more prominent clinical manifestations, the rate of TAIRS diagnosis in female patients is higher than in males.

The patient in this study was a young woman who was hospitalized with a menstrual disorder for 4 years. The patient was nonobese and demonstrated severe hyperinsulinemia, hyperandrogenism, diabetes, menstrual frequency reduction, and polycystic ovaries while showing no signs of acanthosis nigricans. PCOS is a common endocrine metabolic disease, mainly manifested as hyperandrogenemia, menstrual abnormalities, and ovarian polycystic changes, which may be combined with IR and acanthosis nigricans [8]. Baseline 17-OHP less than 2 ng/ml may rule out the diagnosis of congenital adrenal hyperplasia [8]. Our patient was initially diagnosed with PCOS after completion of the relevant examinations and was treated with metformin. After 1 year of follow-up, the clinical manifestations and laboratory indicators of IR and hyperandrogenism were not significantly improved. Multiple examinations showed abnormally elevated insulin levels. Considering that the previous diagnosis might have been incorrect, whole-genome exon sequencing of the patient and her parents was performed. The results showed that exon 20 of the *INSR* gene (NM_000208) in the patient and her father had a heterozygous missense mutation, c.3601C>T, p.R1201W. TAIRS was definitively diagnosed in the patient. Subsequently, sequencing of the same site was performed for the patient’s three brothers; her eldest and elder brothers were also found to have the heterozygous missense mutation p.R1201W. The heterozygous missense mutation c.3601C>T in exon 20 of the *INSR* gene causes the arginine at amino acid position 1201 in the encoded protein to be mutated to tryptophan. This mutation has been confirmed in previous reports to lead to decreased affinity of INSR for insulin and downregulation of tyrosine kinase activity [9, 10].

TAIRS usually demonstrates autosomal dominant inheritance but has demonstrated autosomal recessive inheritance in a few cases. Most patients with TAIRS have only one *INSR* allele, which is mutated, resulting in the production of INSR that does not properly transduce signals. This phenomenon is known as *dominant negative mutation*; that is, these mutated signal transduction proteins interfere with or inhibit the action of wild-type signal transduction proteins in the same cell [11, 12]. Therefore, despite a significant increase in the blood insulin level, the presence of defective receptors prevents the wild-type ones from functioning normally. Severe IR leads to impaired glucose regulation and eventually to diabetes. After female patients enter puberty, severe hyperinsulinemia stimulates the secretion of ovarian androgen, which causes primary amenorrhea, polycystic ovaries, and other manifestations [10]. Even with the same type of mutation at the same site of the *INSR* gene, the IR level is usually relatively mild in male patients [13], as was true in the male patients in the family reported in this article. The female proband of the first TAIRS family reported in China also demonstrated a much more severe degree of IR than did her father [14]. Therefore, a sex bias is observed in the rate of diagnosis.

Currently, TAIRS still has no cure. Treatment is aimed at preventing the long-term complications of diabetes and improving hyperandrogenism [15]. Although most patients are not obese, maintaining a healthy body weight and BMI within the desirable range is important for controlling the blood glucose level. At an early stage of the disease, postprandial hypoglycemia may be the main symptom, and acarbose can reduce postprandial blood glucose fluctuations and postprandial hypoglycemia, which is secondary to hyperinsulinemia. Subsequently, the insulin sensitizers metformin and glitazones are employed as important therapeutic strategies, although with limited efficacy. As diabetes progresses, the use of large doses of exogenous insulin is inevitable [7, 16, 17]. Studies have reported that the use of insulin-like growth factor 1 in a short course of treatment is beneficial for the protection of islet β-cells and for controlling blood glucose levels [18].

Female patients should also be concerned with the treatment of hyperandrogenism to minimize the effects of hyperandrogenemia, such as hirsutism, acne, and amenorrhea. Commonly used drugs include cyproterone acetate, flutamide, and spironolactone. Laser treatment is very effective in improving hirsutism, although it has the risk of causing skin scars [16].

This paper reports a family with TAIRS, which was clearly diagnosed by gene sequencing, and reviews the etiology, clinical manifestations, diagnosis, and treatment strategies of TAIRS through a review of the related literature. It is expected that this study may improve the understanding of this syndrome by the majority of endocrinologists and may help clinicians make a correct etiological diagnosis, start targeted treatment in similar patients as early as possible, and provide accurate genetic counseling to patients’ families.

## Data Availability

Not applicable.

## Abbreviations

17-OHP: 17-hydroxyprogesterone
BMI: Body mass index
FSH: Follicle-stimulating hormone
INSR: Insulin receptor
IR: Insulin resistance
LH: Luteinizing hormone
OGTT: Oral glucose tolerance test
PCOS: Polycystic ovary syndrome
TAIRS: Type A insulin resistance syndrome
